# Dealing with anorexia nervosa among children and adolescents within a regional area

**DOI:** 10.1186/2050-2974-3-S1-O50

**Published:** 2015-11-23

**Authors:** Benjamin Hansen, Paul Sutton, Allahna Heywood, Veronica Stanganelli, Annette Howe, Tanya Dodman

**Affiliations:** 1 CYMHS, Queensland Health, Mackay, Australia

## Introduction

Anorexia has the highest death rate of any psychiatric disorder (15-20%). Research evidence suggests that inpatient treatment is correlated with expensive cost and poor long term outcomes.

## Objectives

To evaluate the implementation of 10 years of family-based treatment for anorexia nervosa.

## Methods

Patients were treated by an interdisciplinary team including: Paediatrician, Child Psychiatrist, social workers, psychologists and nurses with relevant training in Maudsley model.

The average number of bed-days over the 10 year-period prior to the Maudsley approach was 102.8. The average number of patients admitted each year was 3.2 with average length of stay of 32 days per patient.

## Results

The implementation of this approach has been dependent upon an excellent stakeholders' relationship. We achieved 77% reduction in the average number of bed-days; 37% reduction on the admissions, 62% less length of stay and long-term positive outcomes.

## Conclusions

Family-based treatment appears to be the most efficacious treatment for Youth Anorexia Nervosa despite of the scarce resources within Mackay region.

